# Environmental occurrence of *optrA*-mediated linezolid resistance in *Enterococcus* isolates and genomic insights into *Enterococcus faecium* ST54 co-harboring *optrA*, *poxtA*, and *cfr*(D) genes

**DOI:** 10.1007/s11274-026-04786-4

**Published:** 2026-01-31

**Authors:** Lucas David Rodrigues Dos Santos, João Pedro Rueda Furlan, Rafael da Silva Rosa, Micaela Santana Ramos, Letícia Franco Gervasoni, Eduardo Angelino Savazzi, Teresa Nogueira, Eliana Guedes Stehling

**Affiliations:** 1 Department of Clinical Analyses, Toxicology and Food Science, School of Pharmaceutical Sciences of Ribeirão Preto, University of São Paulo, Av. do Café, S/N, Monte Alegre, Ribeirão Preto, São Paulo Brazil; 2 Department of Pharmaceutical Sciences, Health Sciences Center, Federal University of Paraíba, João Pessoa, Paraíba Brazil; 3 Environmental Company of the State of São Paulo, Ribeirão Preto, São Paulo Brazil; 4 National Institute for Agrarian and Veterinary Research (INIAV), I.P., Vila do Conde, 4485-655 Portugal; 5 Centre for Ecology, Evolution and Environmental Changes (cE3c), Global Change and Sustainability Institute (CHANGE), Faculty of Sciences, University of Lisbon, Lisbon, 1749-016 Portugal

**Keywords:** Brazil, Enterococci, Linezolid, Multidrug resistance, Plasmid, Water

## Abstract

Linezolid-resistant *Enterococcus* species have emerged worldwide, with resistance mainly driven by plasmid-borne *optrA*,* poxtA*, and *cfr* genes. While the *optrA* gene has been increasingly identified in humans and animals, its presence in the environmental sector remains poorly studied, especially in South America. This study aimed to investigate and characterize linezolid resistance genes in isolates of *Enterococcus faecium* and *Enterococcus faecalis* obtained from aquatic ecosystems in 51 cities in the state of São Paulo, Brazil. Phenotypic, molecular, and genomic analyses were used for this proposal. Accordingly, 181 *Enterococcus* isolates were obtained, with 67 (37%) harboring the *optrA* gene. Most isolates exhibited multidrug resistance, and the minimum inhibitory concentration to linezolid ranged from 0.5 to > 64 mg/L. Several virulence genes and plasmid replicons were observed, with *gelE* and *rep*_9_ being most prevalent, respectively. Ten isolates co-harbored the *optrA* and *poxtA* genes and belonged to the known sequence type (ST) 1221 (*E. faecium*) and ST283, ST253, ST234, and ST1230 (*E. faecalis*), as well as to the new ST3018, ST3022, ST3026, and ST3027 (*E. faecium*), and ST2126 (*E. faecalis*). Moreover, one *E. faecium* isolate (EW1587) carried *optrA*,* poxtA*, and *cfr*(D) genes and, therefore, was submitted to genomic characterization. Isolate EW1587 belonged to ST54 and was closely related to an animal-derived Brazilian strain. In silico analysis predicted that *optrA*,* poxtA*,* and cfr*(D) genes were plasmid-borne, whereas in vitro stability tests demonstrated that these genes remained stable for 30 days. These results highlight the environmental spread of transferable oxazolidinone resistance genes, with *E. faecium* ST54 co-carrying *optrA*,* poxtA*, and *cfr*(D) genes. Therefore, continuous monitoring is essential to fully elucidate the mechanisms driving the spread and evolution of linezolid resistance across environmental reservoirs.

**Supplementary Information:**

The online version contains supplementary material available at 10.1007/s11274-026-04786-4.

## Introduction

Enterococci are resilient bacteria found as commensal organisms in the gut and have been utilized as indicators for monitoring fecal contamination in the environment and food products (Shen et al. 2023). Among enterococci species, *Enterococcus faecium* and *Enterococcus faecalis* have been reported causing healthcare-associated infections worldwide (Monteiro et al. 2023). These pathogens pose significant health risks to immunocompromised individuals, causing severe conditions such as bacteremia, pneumonia, urinary tract infections, and meningitis (Ngbede et al. 2023; Al Rubaye et al. 2024). Notably, human diseases caused by these species have become increasingly difficult to treat due to intrinsic and acquired resistance to clinically important antimicrobial agents (Yi et al. 2022; Bender et al. 2024). Indeed, antimicrobial-resistant enterococci have emerged rapidly, with vancomycin-resistant *E. faecium* (VRE) classified as a global priority-resistant bacteria by the World Health Organization (Jesudason 2024).

Linezolid, a synthetic antimicrobial agent, was approved for clinical use by the United States Food and Drug Administration (FDA) as the first member of the oxazolidinone class. (Hashemian et al. 2018). It has been widely employed to treat infections caused by multidrug-resistant and vancomycin-resistant enterococci and is considered a last-resort therapeutic option (Ni et al. 2023). However, linezolid-resistant *Enterococcus* has also emerged, posing serious challenges to public health. Accordingly, linezolid resistance is primarily mediated by mutations in the central loop of domain V of the 23S rRNA gene, particularly the G2576U and G2505U mutations according to *Escherichia coli* numbering, which have been the most prevalent and well-characterized ones (Wilson et al. 2008).

Furthermore, linezolid resistance also occurs by dissemination of transferable oxazolidinone resistance genes through mobile genetic elements, including conjugative plasmids, integrative and conjugative elements (ICEs), which facilitate their rapid spread among different strains and environments (Strateva et al. 2025). Among these genes, *optrA* is the most frequently reported worldwide (Shen et al. 2023). This gene encodes an ATP-binding cassette (ABC-F) protein that protects the bacterial ribosome, mediating resistance to both oxazolidinones and phenicols (Almeida et al. 2020). The *optrA* gene also confers resistance to tedizolid, which was approved by the FDA in 2014 as a second-generation oxazolidinone. (Fu et al. 2024). To date, several *optrA* variants have been described, including OptrA V1, V2, V3, V4, V5, V6, V7, V8, V9, V10, V11, V12, and V13. In Brazil, some of these variants, such as V9, V12, and V13, have already been reported in *Enterococcus faecalis*, highlighting the genetic diversity and geographical spread of this resistance determinant (Almeida et al. 2020). Another important gene is *poxtA*, which encodes an ABC-F ribosomal protection protein that reduces susceptibility to oxazolidinones and phenicols (Antonelli et al. 2018; Crowe-McAuliffe et al. 2022). Additionally, the *cfr* gene encodes a methyltransferase that methylates nucleotide A2503 of the 23S rRNA after transcription. This modification confers resistance not only to oxazolidinones but also to phenicols, lincosamides, pleuromutilins, and streptogramin A (denominated PhLOPSA phenotype) (Deshpande et al. 2015; Schwarz et al. 2021).

Recently, novel variants of the *cfr* gene, such as *cfr*(B) and *cfr*(D), have been identified at the human-animal-environmental interface (Guerin et al. 2020; Ruiz-Ripa et al. 2020). While the *cfr*(D) gene is not as well-characterized as other variants, it has been identified in clinically relevant *Enterococcus* and *Staphylococcus* species across various continents (Gao et al. 2022; Kim et al. 2024). Its genetic configuration is often associated with insertion sequences (IS) on conjugative plasmids (Cinthi et al. 2022a, b).

From the anthropogenic perspective, contaminated water sources have contributed to the global evolution and spread of antimicrobial resistance (AMR). In this context, surface waters represent important drivers of AMR, as they harbor a dynamic microbial community influenced by several characteristics (Reddy et al. 2022; Dos Santos et al. 2023). Despite the documented rise in linezolid-resistant enterococci (LRE) in clinical settings, their occurrence, genomic characteristics, and potential role as environmental reservoirs remain poorly understood. Therefore, this study aimed to characterize LRE isolates recovered from surface waters in Brazil using phenotypic, molecular, and genomic approaches.

## Materials and methods

### Environmental samples and bacterial isolation

During a surveillance study to monitor the presence of LRE in aquatic ecosystems, 136 surface water samples were collected from rivers and streams across 51 cities in São Paulo State, Brazil, between July 2021 and January 2022. The sampling points were located in areas exposed to multiple anthropogenic influences, including urban activity, recreational water use, livestock farming, and points containing discharge of domestic and hospital wastewater. For bacterial isolation, each water sample (1 L) was filtered using a sterile membrane filter with a pore size of 0.45 μm, which was then added to plates of Kanamycin Esculin Azide agar (HiMedia, India) supplemented with 4 mg/L of linezolid (Sigma-Aldrich, USA). After that, the plates were incubated for 48 h at 37 °C. Finally, dark (black or brown) colonies, morphologically typical of *Enterococcus* species, were selected and stored at − 80 °C using Brain Heart Infusion broth (Kasvi, Spain) supplemented with 15% glycerol.

### Molecular identification

Genomic DNA of each colony was extracted according to Ramos et al. (2023), with modifications. Specifically, a pre-incubation step with 50 mg/mL of lysozyme (Sigma-Aldrich, USA) at 37 °C for 30 min was included to enhance bacterial cell wall lysis and improve DNA extraction efficiency. The identification was performed by conventional polymerase chain reactions (PCR) using a genus-specific gene for the *Enterococcus* genus and species-specific genes for *E. faecium* and *E. faecalis* (Supplementary Table S1). Furthermore, the Sanger sequencing of the 16S rRNA gene was also performed, and the sequences were analyzed using the blastn suite of BLAST^®^ (https://blast.ncbi.nlm.nih.gov/Blast.cgi).

### Antimicrobial susceptibility testing and high-level AMR

Antimicrobial susceptibility was initially determined using the disk diffusion method. The antimicrobials tested were linezolid, vancomycin, teicoplanin, ampicillin, imipenem, tetracycline, doxycycline, minocycline, ciprofloxacin, levofloxacin, norfloxacin, erythromycin, chloramphenicol, fosfomycin, nitrofurantoin, and rifampicin (Cecon, Brazil). The broth microdilution method was performed to determine the minimum inhibitory concentration (MIC) for linezolid and vancomycin with concentrations ranging from 1 to 256 µg/mL. The susceptibility results for all antimicrobials were interpreted according to the Brazilian Committee on Antimicrobial Susceptibility Testing (BrCAST; v.10.0, 2023), except for tetracycline, doxycycline, minocycline, chloramphenicol, rifampicin, fosfomycin, and erythromycin, for which the Clinical and Laboratory Standards Institute guidelines (CLSI, M100, 30th, 2020) were used. The enterococci isolates were classified as multidrug-resistant (MDR) using the criteria of Magiorakos et al. (2012). The high-level ciprofloxacin resistance (HLCR) and high-level aminoglycoside resistance (HLAR) were evaluated by agar dilution method using ciprofloxacin (64 µg/mL) and gentamicin (500 µg/mL)/streptomycin (2000 µg/mL), respectively (CLSI, M100, 30th, 2020; Leavis et al. 2006; Adhikari et al. 2010).

### Detection of antimicrobial resistance genes (ARGs), virulence genes, and plasmid replicons

Conventional PCR was used in this step. Genes that confer resistance to linezolid [*optrA*, *poxtA*, *cfr*, *cfr*(B), and *cfr*(D)], aminoglycosides [*aph(2’’)-Ib*,* ant(4’)-Ia*,* aac(6’)-Ie-aph(2’’)-Ia*, *ant(6’)-Ia*, *aph(3’)-IIIa*, *aph(2’’)-Id*, and *aph(2’’)-Ic*], tetracyclines [*tet*(K), *tet*(L), *tet*(M), and *tet*(O)], vancomycin (*vanA* and *vanB*), and erythromycin [*erm*(A), *erm*(B), *erm*(C), and *mefAE*] were screened. In addition, virulence genes encoding adhesin to collagen (*ace*), gelatinase (*gelE*), enterococcal surface protein (*esp*), aggregation substances (*agg*), and hyaluronidase (*hyl*), as well as plasmid replicons (*rep*-_1_ to *rep*-_19_), were also investigated. All primers and conditions are described in the Supplementary Tables S2, S3, and S4.

### Identification of mutations in the 23S rRNA

The amplified region corresponds to domain V of the 23S rRNA gene and was analyzed in all *optrA*-positive isolates. The 23S rRNA region was amplified by PCR using 48 °C annealing temperature and the primers 5′-GACGGAAAGACCCCATGG-3′ and 5′-ACACTTAGATGCTTT-3′, which target nucleotide positions 2049 to 2767 and result in a 718-bp fragment (Prystowsky et al. 2001). The amplicons were purified using the Illustra™ GFX™ PCR DNA and Gel Band Purification Kit (GE Healthcare, UK) and sequenced using the BigDye Terminator Cycle Sequencing Kit (Applied Biosystems, USA) on an ABI 3500xL Genetic Analyzer. The obtained sequences were analyzed by Geneious Prime^®^ 2025.0.2 using 23S rRNA regions from *E. faecalis* V583 (GenBank: NC_004668.1) and *E. faecium* DO (GenBank: CP003583) as references.

### Multilocus sequence typing (MLST)

The housekeeping genes used for *E. faecium* were *adk*,* atpA*,* ddl*,* gyd*,* gdh*,* purK*, and *pstS*, while for *E. faecalis* were *gdh*,* pstS*,* gyd*,* gki*,* xpt*, *aroE*, and *yqiL*. The genes were amplified and sequenced using the primers and conditions described in the Supplementary Table S5. The results were analyzed using the PubMLST databases for *E. faecalis* (https://pubmlst.org/organisms/enterococcus-faecalis) and *E. faecium* (https://pubmlst.org/organisms/enterococcus-faecium).

### Whole-genome sequencing (WGS) and analysis

Genomic DNA extraction was performed using the PureLink™ Genomic DNA Mini Kit (Thermo Fisher Scientific, USA), and the WGS was conducted on the Illumina MiSeq platform (Illumina Inc., USA). Subsequently, the draft genome was *de novo* assembled using SPAdes v.3.15.2 (https://github.com/ablab/spades) and annotated using RAST (https://rast.nmpdr.org/rast.cgi*).* To identify the acquired ARGs, genes, and mutations leading to linezolid resistance, acquired virulence genes, plasmid replicons, and sequence type (ST), we used the bioinformatic tools ResFinder v.4.7.2, LRE-Finder v.1.0, VirulenceFinder v.2.0, PlasmidFinder v.2.0, and MLST v.2.0, respectively, with default parameters available from the Center for Genomic Epidemiology (http://www.genomicepidemiology.org/*).* Plasmid assembly was predicted using RFPlasmid (http://klif.uu.nl/rfplasmid/*)* and further refined using combined strategies involving BLASTn and Geneious Prime^®^ v.2022.2.2 (Biomatters Ltd.). The analysis of insertion sequence elements was performed using ISfinder (https://www-is.biotoul.fr/index.php*).*

### Comparative analysis of ST54 isolates

A phylogenetic tree was constructed based on single-nucleotide polymorphism (SNP) analysis to investigate the genetic relatedness among *E. faecium* isolates belonging to ST54. The isolate EW1587 (this study) and public genomes (*n* = 25) available on the PathogenWatch database (https://pathogen.watch/) on May 1, 2025, were included in this analysis. SNP calling and phylogenetic inference were performed using the CSI Phylogeny v.1.4 (https://cge.food.dtu.dk/services/CSIPhylogeny/). Based on analyses performed using QUAST v.5.3.0 and CheckM v.1.2.3, the 11F10-MSG5009 genome (GenBank: NZ_NGMA00000000.1) was selected as reference for ST54. The phylogenetic tree was visualized by iTOL v.7 (https://itol.embl.de/). The plasmid reconstruction was predicted by PLACNETw (Vielva et al. 2017).

### Plasmid stability test

Isolate *E. faecium* EW1587 co-harboring the *optrA*, *poxtA*, and *cfr*(D) genes was subjected to a plasmid stability assay for 30 days following the protocol described by Fukuda et al. (2024), with modifications. The EW1587 isolate was first inoculated into 5 mL of Brain Heart Infusion (BHI) broth (Kasvi, Spain) and incubated overnight at 37 °C. Then, 5 µL of this culture was transferred into fresh BHI, corresponding to a 1:1000 dilution. Subcultures were maintained for 30 consecutive days under three conditions: (i) room temperature, (ii) static incubation at 37 °C, and (iii) incubation at 37 °C with shaking at 124 rpm. Samples were seeded daily onto plates of BHI agar (Oxoid, UK) supplemented with linezolid (4 mg/L) and without antimicrobial, which were incubated at 37 °C for 24 h. Approximately ten colonies per plate were randomly selected for PCR-based screening of *optrA*, *poxtA*, and *cfr*(D) genes and MIC determination for linezolid.

## Results

### *Enterococcus* isolates harboring transferable oxazolidinone resistance genes

**Fig. 1:**
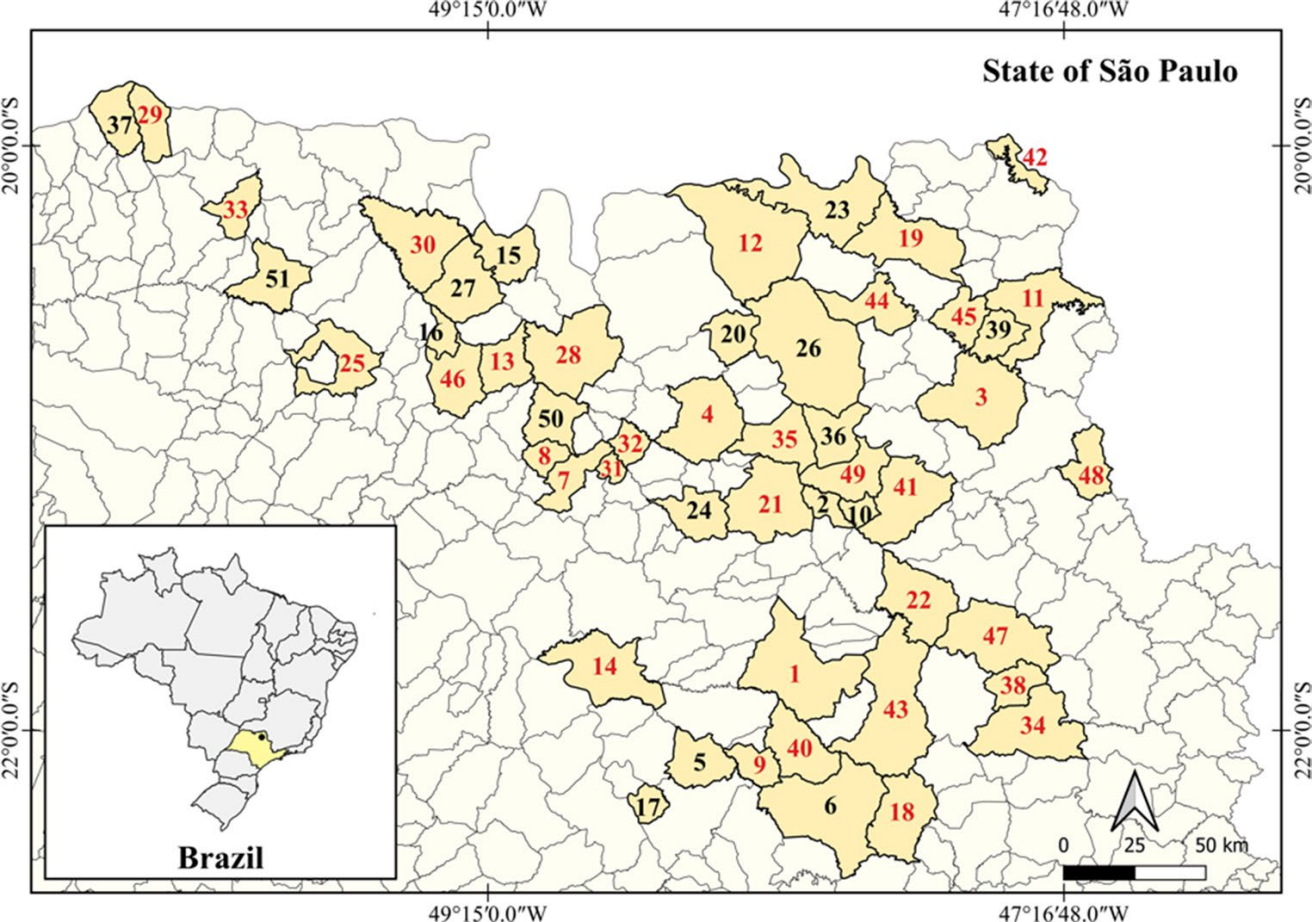
Map showing the 51 cities where samples were collected. The Brazilian cities are as follows: 1: Araraquara; 2: Barrinha; 3: Batatais; 4: Bebedouro; 5: Bocaina; 6: Brotas; 7: Catanduva; 8: Catiguá; 9: Dourado; 10: Dumont; 11: Franca; 12: Guaíra; 13: Guapiaçu; 14: Ibitinga; 15: Icém; 16: Ipiguá; 17: Itapuí; 18: Itirapina; 19: Ituverava; 20: Jaborandi; 21: Jaboticabal; 22: Luís Antônio; 23: Miguelópolis; 24: Monte Alto; 25: Monte Aprazível; 26: Morro Agudo; 27: Nova Granada; 28: Olímpia, 29: Ouroeste; 30: Palestina; 31: Palmares Paulista; 32: Paraíso; 33: Pedranópolis; 34: Pirassununga; 35: Pitangueiras; 36: Pontal; 37: Populina; 38: Porto Ferreira; 39: Restinga; 40: Ribeirão Bonito; 41: Ribeirão Preto; 42: Rifaina; 43: São Carlos; 44: São José da Barra; 45: São João da Bela Vista; 46: São José do Rio Preto; 47: Santa Rita do Passa Quatro; 48: Santo Antônio da Alegria; 49: Sertãozinho; 50: Tabapuã; 51: Votuporanga. The numbers highlighted in red indicate the locations where *optrA*-positive enterococci were obtained

A total of 181 *Enterococcus* spp. isolates were obtained, of which 67 (37%) harbored the *optrA* gene. These isolates were identified as *E. faecalis* (*n* = 52, 77%) and *E. faecium* (*n* = 15, 23%) (Supplementary Table S6). Interestingly, five isolates from each species (*E. faecalis*: EW1636, EW1662, EW1668, EW1670, and EW1672; *E. faecium*: EW1637, EW1638, EW1639, EW1681, and EW1682) co-carried the *optrA* and *poxtA* genes. Furthermore, the *E. faecium* isolate EW1587 harbored the three transferable oxazolidinone resistance genes *optrA*, *poxtA*, and *cfr*(D) (Fig. 1).

Notably, different isolates carrying the *optrA* gene were obtained from the same river systems across different municipalities, indicating the spread of linezolid-resistant isolates through waterways. Specifically, *E. faecalis* isolates EW1648, EW1649, and EW1650 were recovered from the São Domingos Stream in three different cities. Similarly, *E. faecium* isolates EW1670 and EW1672 originated from the Mogi-Guaçu River in two distinct locations. Furthermore, *E. faecium* isolates EW1681 and EW1682, both carrying *optrA* and *poxtA* genes, were isolated from the Jacaré-Guaçu River (Supplementary Table S6).

### AMR profiles, HLAR, and HLCR

Antimicrobial susceptibility testing was performed exclusively on the 67 *optrA*-positive isolates. All isolates showed resistance to imipenem, and 94% were resistant to linezolid. In *E. faecalis*, the MICs for linezolid ranged from 0.5 mg/L to 128 mg/L, whereas in *E. faecium*, the MICs for linezolid ranged from 2 mg/L to 128 mg/L. Regarding the MICs for vancomycin, *E. faecalis* exhibited values ranging from 1 mg/L to 8 mg/L, while in *E. faecium*, MICs ranged from 1 mg/L to 4 mg/L. Overall, all *E. faecalis* isolates were classified as MDR, presenting elevated resistance rates to tetracycline (88%), fluoroquinolones (63%), and chloramphenicol (69%). In contrast, lower resistance frequencies were identified for nitrofurantoin (19%) and ampicillin (3%). For *E. faecium*, 46% of isolates were classified as MDR; nonetheless, these isolates were resistant to at least four distinct antimicrobial classes. The most frequent resistance rates were observed for rifamycins (86%), fosfomycin (66%), and fluoroquinolones (40%). High-level gentamicin resistance (HLGR) was identified in 29 isolates (43%), while high-level streptomycin resistance (HLSR) was detected in 23 isolates (34%). Notably, HLGR + HLSR was found in 21 isolates (31%). Besides, HLCR was detected in six *E. faecalis* isolates (9%) (Supplementary Table S7).

### Enterococci harboring ARGs, virulence genes, and plasmid replicons

**Fig. 2:**
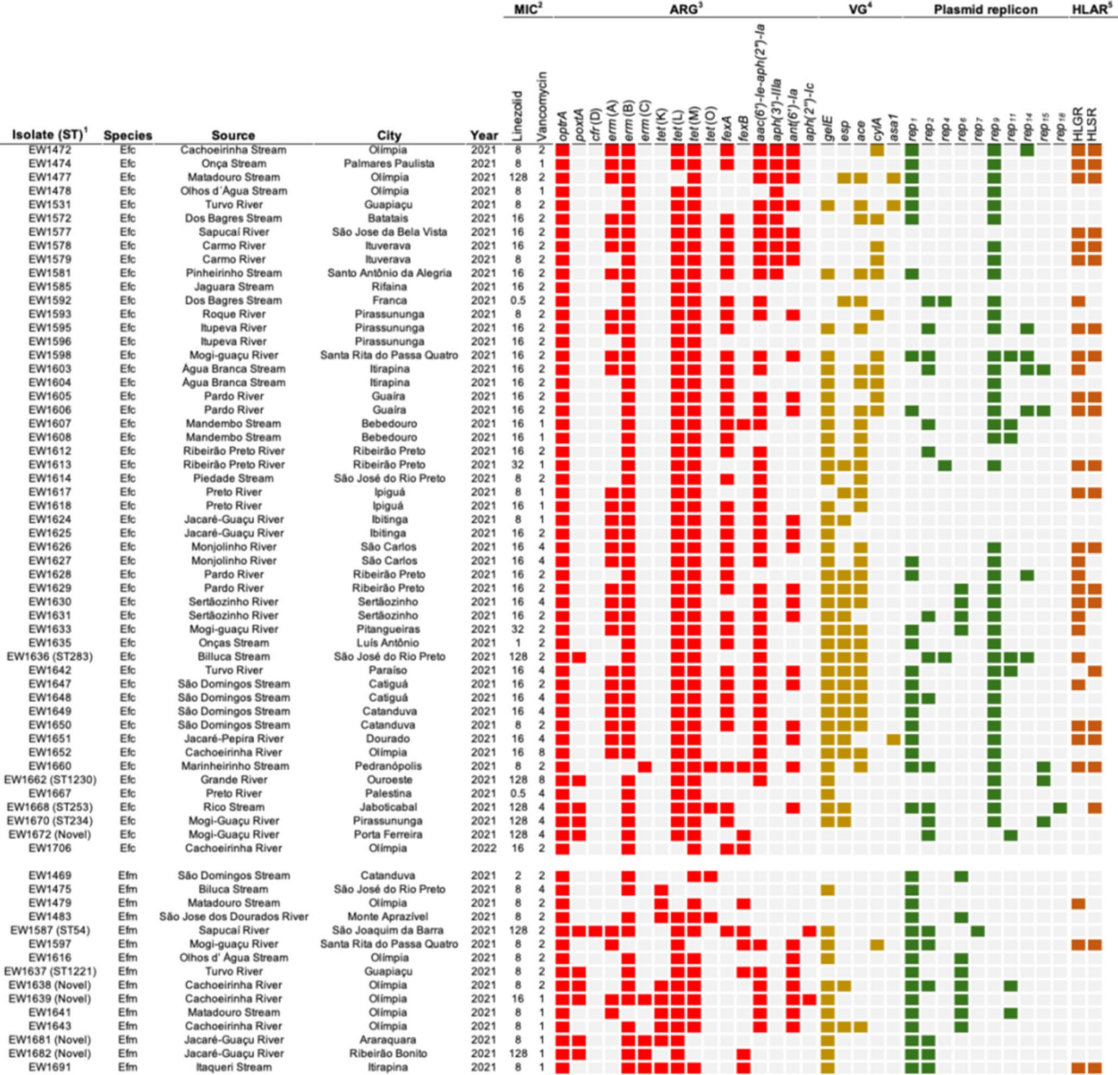
Overview of *E. faecalis* (Efc) and *E. faecium* (Efm) isolates obtained in this study. Colored squares indicate the presence of specific genetic determinants. ^1^ Sequence type (ST). ^2^ Minimum inhibitory concentration (MIC). ^3^ Antimicrobial resistance gene (ARG). ^4^ Virulence gene (VG). ^5^ High-level aminoglycoside resistance (HLAR); High-level gentamicin resistance (HLGR); and high-level streptomycin resistance (HLSR)

In addition to transferable oxazolidinone resistance genes, ARGs to other antimicrobial classes were also detected. In *E. faecalis* isolates, the most prevalent ARGs were *tet*(M) (*n* = 52, 100%), *tet*(L) (*n* = 50, 96%), *erm*(B) (*n* = 51, 98%), and *aac(6′)-Ie-aph(2″)-Ia* (*n* = 40, 77%) (Fig. 2). Among *E. faecium* isolates, the most frequently detected genes were *tet*(L) (*n* = 12, 80%), *tet*(M) (*n* = 11, 73%), *tet*(K) (*n* = 9, 60%), and *erm*(B) (*n* = 12, 80%). Only *E. faecium* isolates harbor *tet*(K), and *aph(2’’)-Ic* genes. Furthermore, the *aac(6′)-Ie-aph(2″)-Ia* gene was found in 25 HLAR-positive isolates (83%), while the *aph(3′)-IIIa* gene was detected in four HLAR-positive isolates (12%) (Fig. 2).

Virulence genotyping revealed that the *gelE* gene, which encodes a gelatinase, was the most detected virulence factor (*n* = 48, 71%), followed by the *ace* gene (encoding collagen adhesin) detected in 32 isolates (47%), and the *esp* gene (encoding enterococcal surface protein) found in 23 isolates (34%). The cytolysin gene *cylA* was found in 12 isolates (18%), while the aggregation substance gene *asa1* was the least prevalent, as it was detected in three isolates (4%). Several plasmid replicons were observed, with 56 (83%) isolates harboring at least one *rep*-type. The most prevalent families were *rep*_9_ (*n* = 41, 61%), *rep*_1_ (*n* = 37, 55%), *rep*_2_ (*n* = 19, 28%), and *rep*_6_ (*n* = 12, 18%). Notably, 46 isolates (68%) carried two or more *rep*-types. Species-specific analysis revealed distinct plasmid profiles. *E. faecalis* isolates showed greater plasmid diversity, with *rep*_9_ being the most frequent (*n* = 39; 75%), but 11 isolates (21%) did not harbor the *rep*-type searched. In contrast, all *E. faecium* isolates harbored plasmid replicons and exhibited a more conservative profile, with *rep*_1_ detected in all isolates, followed by *rep*_6_ (*n* = 8, 53%) and *rep*_2_ (*n* = 6, 40%) (Fig. 2).

### Plasmid stability

In vitro stability experiments showed that isolate EW1587 maintained *optrA*, *poxtA*, and *cfr*(D) genes and plasmid replicons for 30 days, allowing their long-term persistence.

### Analysis of 23S rRNA mutations

The known mutations A2571G and G2595C associated with linezolid resistance were identified in two *E. faecalis* isolates (EW1472, EW1477) and one *E. faecium* isolate (EW1587). In addition to these well-established mutations, several other nucleotide changes were detected in the 23S rRNA gene. However, these additional mutations have not yet been associated with linezolid resistance in *Enterococcus* species. Accordingly, among the 52 *E. faecalis* isolates, 11 (21%) carried at least one of these uncharacterized mutations, with T2131A, G2134A, C2136T, G2153A, A2154T, T2179C, T2182C, A2189G, A2190C, C2192A, and A2210G being the most prevalent. On the other hand, *E. faecium* isolates (*n* = 7, 46%) presented the predominant A2211G mutation (Supplementary Table S8).

### Enterococcal clones

MLST analysis was performed for all *E. faecium* and *E. faecalis* isolates that co-carry the *optrA* and *poxtA* genes. Among *E. faecalis* isolates, four were successfully assigned to known sequence types: ST283 (EW1636), ST253 (EW1668), ST234 (EW1670), and ST1230 (EW1662). In *E. faecium*, only one isolate (EW1637) was typed as ST1221. For the remaining isolates, *E. faecalis* (EW1672) and *E. faecium* (EW1638, EW1639, EW1681, and EW1682), the MLST scheme yielded novel allelic combination profiles that were signed as new sequence types as follows: ST2126 (*E. faecalis*) and ST3018, ST3022, ST3026, and ST3027 (*E. faecium*) (Supplementary Table S9).

### Genomic insights into *E. faecium* ST54 co-harboring *optrA*, *poxtA*, and *cfr*(D) genes

The EW1587 isolate carried three transferable oxazolidinone resistance genes [*optrA*, *poxtA*, and *cfr*(D)] (GenBank: JBQEQP000000000). In addition, this isolate harbored other ARGs, including the acquired *erm*(B), *fexB*, *fexA*, *tet*(M), and *tet*(L), as well as the intrinsic *aac(6’)-I*, *msr(C)*. Furthermore, the OptrA_13 variant (Tyr176Asp and Gly393Asp) was identified. No known mutations were found in GyrA and ParC, while 13 amino acid substitutions (V24A, S27G, R34Q, G66E, E100Q, K144Q, T172A, L177I, A216S, T324A, N496K, A499T, and E525D) were detected in penicillin-binding protein 5 (PBP5) and are known to be related to the PBP5-R (resistant) isoform. Virulome analysis revealed the presence of various virulence genes, including *bepA*, *ccpA*, *empA*, *empB*, *empC*,* fms1*, *fms13*, *fms14*, *fms15*, *fms16*, *fms17*, *fms19*, *fms20*, *fms21*, *fnm*, *gls20*, *gls33*, *glsB1*, *sagA*, *scm*, and *sgrA.* These genes have been mainly associated with biofilm formation, hydrolase enzymes, surface proteins, and adhesive matrix components. The plasmid replicons *rep*_*29*_, *rep*_*1*_, *rep*_*2*_, *rep*_*7a*_, *repUS43*, and *repUS15* were found.

**Fig. 3:**
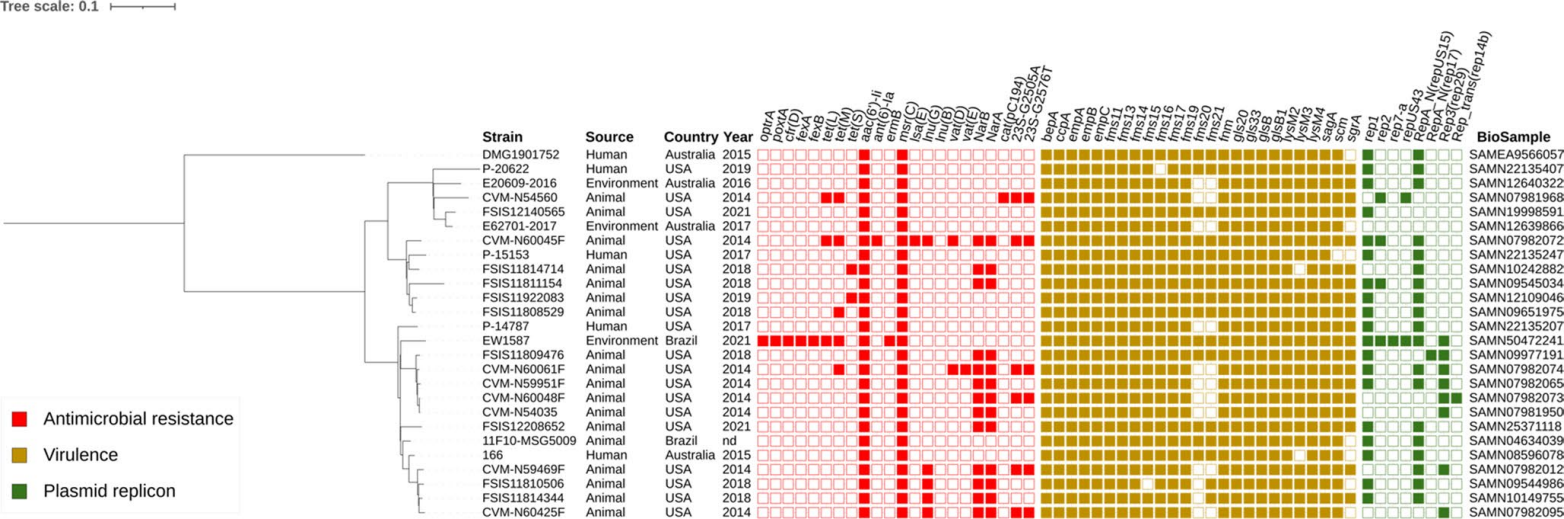
SNP-based phylogenetic tree of *E. faecium* ST54 genomes. Colored squares indicate the presence of specific genetic determinants as follows: red color for antimicrobial resistance; dark yellow color for virulence genes; and green color for plasmid replicons. United States (USA). Not determined (nd). The tree was rooted at the midpoint

Isolate EW1587 belonged to ST54. SNP differences among globally distributed ST54 genomes ranged from 5 to 2034. EW1587 formed an external branch relative to ST54 isolates from animals and humans in Australia, Brazil, and the United States, differing by 206–289 single-nucleotide polymorphisms (SNPs) (Fig. 3). The environmental isolate EW1587 showed a difference of 206 SNPs from the animal-derived isolate 11F10-MSG5009 from Brazil, indicating a distant common ancestor and supporting long-term diversification within the ST54 lineage rather than recent transmission. Across all ST54 genomes, a broad resistome, a conservative virulome, and diverse plasmid replicons were identified. Notably, none of the ST54 genomes carried oxazolidinone resistance genes, suggesting that acquisition of such determinants likely occurred by recent horizontal gene transfer with local microbial communities.

The location of the *optrA*,* poxtA*, and *cfr*(D) genes was predicted in silico as plasmid-associated based on short-read data. Moreover, by using PLACNETw, plasmid-associated contigs were identified through the detection of relaxase and/or replication elements, and the *optrA* gene was detected on the same contig as *erm*(B) and *rep*_*7a*_. Although *poxtA* and *cfr*(D) genes were located singly on different contigs, they were connected by solid lines, suggesting they belong to the same scaffold and are likely part of the same genetic environment. Additionally, *tet*(M) and *tet*(L) genes were found on another contig grouped within the same plasmid cluster (Supplementary Fig. S1). The genetic context of *optrA* from this study was similar to that found in the pL14 plasmid (GenBank: CP043725) of *E. faecalis* ST330 from swine in Brazil. Accordingly, the *araC* gene from the core *araC*-*hp1*-*optrA* of the EW1587 isolate was truncated (Δ*araC* 125 bp) when compared with that of the pL14 plasmid (*araC* 1,155 bp).

## Discussion

To address the environmental spread of linezolid resistance in Latin America, studies conducted in Brazil have provided valuable insights. Environmental analyses of water and soil samples have identified *E. faecium* and *E. casseliflavus* isolates resistant to linezolid, although these isolates had exhibited a low prevalence of transferable resistance genes (Dos Santos et al. 2021, 2023). Similar findings have been reported in *Enterococcus* isolates from recreational waters, where linezolid resistance was observed despite the absence of detectable transferable resistance genes (Santiago et al. 2024). Nevertheless, metagenomic analyses of DNA extracted from treated sewage samples revealed the presence of the *optrA* and *poxtA* genes (Dias et al. 2020). Based on the available data, the widespread dissemination of linezolid resistance genes in environmental sources appears to remain limited. In contrast to these reports, our findings reveal a high frequency of transferable oxazolidinone resistance genes in surface water-derived enterococci, highlighting the environmental compartment as a relevant and underexplored reservoir.

Data on the prevalence of linezolid-resistant *Enterococcus* in Brazilian hospitals are also scarce. A low resistance rate (2.9%) was reported and associated with the G2576T mutation in the 23S rRNA gene in clinical isolates (Jones et al. 2013). Similarly, a study conducted in a hospital in Curitiba, Paraná (southern Brazil), found that all VRE isolates were susceptible to linezolid (Vasconcelos et al. 2024). Therefore, the data suggest that linezolid resistance in *Enterococcus* remains relatively low in Latin America, which is predominantly associated with chromosomal mutations rather than mediated by plasmids. Conversely, in the present study, a high frequency of *optrA* gene detection was observed, including isolates carrying multiple oxazolidinone resistance genes in environmental samples, suggesting that these genes may be maintained outside clinical settings.

This study identified a high frequency of the *optrA* gene, as well as isolates co-harboring multiple oxazolidinone resistance genes, in environmental samples. The *optrA* gene was described in 2015 and has been found in species other than enterococci, including *Streptococcus suis*, *Streptococcus parasuis*, *Staphylococcus* sp., *Listeria monocytogenes*, *Clostridium perfringens*, and *Vagococcus lutrae* (Shen et al. 2022). Retrospective analyses suggest that *optrA*-positive LRE isolates have been circulating in hospitals worldwide since 2005 (Freitas et al. 2019; Almeida et al. 2020). Accordingly, the *optrA* gene has been considered a major contributor to the rising incidence of LRE (Wang et al. 2015; Schwarz et al. 2021; Freitas et al. 2020). The variant and context of OptrA from the EW1587 isolate were V13 (Tyr176Asp and Gly393Asp) and P3-like, respectively. These characteristics were previously observed in a swine-derived isolate in Brazil (Almeida et al. 2020).

The dispersion of linezolid-resistant *Enterococcus* species has been mainly associated with humans and food-producing animals. Although not approved for farm use, linezolid resistance may be driven by florfenicol and oxytetracycline overuse in livestock and aquaculture (Gharbi et al. 2024). The selective pressure mediated by these antimicrobials may select plasmids co-harboring *optrA*, *tet*(L), *tet*(M), *erm*(A), and *erm*(B) genes (Yoon et al. 2020; McHugh et al. 2022; Dos Santos et al. 2023). Furthermore, insertion sequences (IS1*216E*) and transposons (Tn*554* and Tn*558*) also contribute to the spread of the *optrA* gene. Accordingly, these mobile genetic elements enable horizontal gene transfer across diverse bacterial hosts and environmental niches (Fu et al. 2024; Liu et al. 2024; Yang et al. 2024).

A broad distribution of *rep*-type plasmid genes was observed, with *rep*_9_ being the most prevalent. These findings corroborate previous reports of *rep*_9_ plasmids as harboring the *optrA* in *E. faecalis* (Mikalsen et al. 2015; Zou et al. 2020). This plasmid family includes several pheromone-responsive and conjugative plasmids, including pAD1, which play a central role in the dissemination of antimicrobial resistance determinants (Jensen et al. 2010; Zou et al. 2020). Therefore, the high prevalence of *rep*_9_ identified in this study supports its species-specific association and suggests that pheromone-responsive transfer systems may contribute to the maintenance and spread of the *optrA* gene.

Mutational linezolid resistance was also identified in *optrA*-positive isolates. The well-characterized 23S rRNA mutations were detected as follows: A2571G in *E. faecalis* and G2595C in *E. faecium*. In addition, other mutations outside the commonly reported resistance-associated regions were also observed and may also be related to linezolid resistance, although such reports have so far only been described in *Staphylococcus aureus* and *Mycobacterium smegmatis* (Sander et al. 2002; Wilson et al. 2008; Long et al. 2010; Long et al. 2012). In this context, new point mutations in linezolid resistance determinants should be further explored for their roles in high-level resistance.

While *optrA* and *poxtA* have been extensively reported, emerging variants like *cfr*(D) remain poorly identified. The *cfr*(D) gene was first identified in a clinical *E. faecium* isolate from France in 2015 (Guerin et al. 2020). Despite its structural similarity to other *cfr*-like genes, functional studies have demonstrated that *cfr*(D) alone does not confer linezolid resistance in enterococci (Hu et al. 2022). In contrast, phenotypic resistance has been observed in *Escherichia coli* carrying *cfr*(D), suggesting that its activity may be host-dependent or influenced by specific genetic contexts (Hu et al. 2022). A previous study demonstrated that *cfr*(D) can frequently coexist with the IS*1216*, which facilitates its mobilization through the formation of transposable units with variable structures (Gao et al. 2025). Consequently, the presence of *cfr*(D) in diverse bacterial species further supports its potential for interspecies transfer (Zhu et al. 2021). In this study, we report the first identification of *cfr*(D) in South America (Supplementary Fig. S2).

The identification of *Enterococcus* species co-carrying transferable oxazolidinone resistance genes is noteworthy. The co-occurrence of *optrA*, *poxtA2*, and *cfr*(D) genes was detected in *E. faecalis* from duck meat in China, *E. gallinarum* of swine origin in Italy, and *E. faecium* from swine in Nigeria. Additionally, clinical isolates of *E. faecalis* and *E. faecium* also harbored these genes (Shen et al. 2023; Ngbede et al. 2023; Coccitto et al. 2022; Torabi et al. 2023). Notably, in the EW1587 isolate, *cfr*(D) is located near *poxtA*, potentially within the same resistance region that can be transmitted at a high rate through horizontal transfer (Cinthi et al. 2022a, b; Nüesch-Inderbinen et al. 2022; Shen et al. 2023). Furthermore, acquired ARGs that confer resistance to other antimicrobials, including macrolides, tetracyclines, and aminoglycosides, as well as substitutions that reduce the binding affinity of PBP5 for ampicillin, have also been identified, highlighting the multidrug resistance phenotype that limits therapeutic options for infections caused by enterococci (Pietta et al. 2014; Novais et al. 2016; Said et al. 2019; Andrea et al. 2025).

The detection of new sequence types among enterococci studied highlights the genetic diversity of the environmental linezolid-resistant isolates. *E. faecalis* ST1230 was singly identified in South Korea from a non-hospitalized patient (PubMLST ID: 2314), whereas ST234 and ST253 have been previously associated with linezolid resistance mediated by the *optrA* gene in human and animal medicine (Mortelé et al. 2024; Gagetti et al. 2023; Nüesch-Inderbinen et al. 2023). By using the PubMLST database, *E. faecalis* ST283 was identified in animals and surface water in Europe and humans from Mexico, the latter harboring the *optrA* gene (Martínez-Ayala et al. 2025).

Among the *E. faecium* isolates, only ST1221 was assigned using the conventional MLST technique. This clone was previously reported in an environmental sample from the United Kingdom (*E. faecium* ID PubMLST: 2962). Genomic analysis identified ST54, with its first genome-associated report in 2014. In Brazil, there is only one genomic report in a green turtle (GenBank: NZ_NGMA00000000.1). In this context, the scarcity of published studies on these *Enterococcus* clones limits our understanding of their diversity and dissemination. Taken together, these findings indicate that environmental enterococci comprise diverse and poorly characterized lineages carrying transferable oxazolidinone resistance genes, supporting the role of surface waters as reservoirs that may contribute to the long-term persistence and evolution of linezolid resistance.

## Conclusion

This study reports the occurrence of transferable oxazolidinone resistance genes in *Enterococcus* species isolated from surface waters in Brazil. The high prevalence of *optrA* in the environment likely results from a multifaceted interplay of factors, including indirect selection pressure from non-linezolid antimicrobials, plasmid-mediated co-resistance, and efficient horizontal gene transfer mechanisms. These findings highlight the environment as an underappreciated reservoir and disseminator of transferable oxazolidinone resistance genes. Therefore, continuous monitoring is essential to comprehensively understand the processes involved in the spread and evolution of linezolid resistance within the environmental sector.

## Supplementary information

Below is the link to the electronic supplementary material.

Supplementary Material 1 (DOCX 1.19 MB)

## Data Availability

Data availabilityThe genome assembly of strain EW1587 was submitted to GenBank under accession number: JBQEQP000000000.
