# Supplementary material for: Environmental occurrence of optrA-mediated linezolid resistance in Enterococcus isolates and genomic insights into Enterococcus faecium ST54 co-harboring optrA, poxtA, and cfr(D) genes
Source: World J Microbiol Biotechnol. 2026 Jan 31;42(2):70. doi: 10.1007/s11274-026-04786-4 (PMC12858486; doi:10.1007/s11274-026-04786-4)
Supplement: Supplementary file 1 — Supplementary Material 1 (DOCX 1.19 MB) [file 11274_2026_4786_MOESM1_ESM.docx]

**Supplementary Material**

**Environmental occurrence of *optrA*-mediated linezolid resistance in *Enterococcus* isolates and genomic insights into *Enterococcus faecium* ST54 co-harboring *optrA*, *poxtA*, and *cfr*(D) genes**

**Authors:**

Lucas David Rodrigues dos Santos^a^, João Pedro Rueda Furlan^a,b^, Rafael da Silva Rosa^a^, Micaela Santana Ramos^a^, Letícia Franco Gervasoni^a^, Eduardo Angelino Savazzi^c^, Teresa Nogueira^d^ and Eliana Guedes Stehling^a,^*

**Affiliations:**

^a^ Department of Clinical Analyses, Toxicology and Food Science, School of Pharmaceutical Sciences of Ribeirão Preto, University of São Paulo, Brazil.

^b^ Department of Pharmaceutical Sciences, Health Sciences Center, Federal University of Paraíba, João Pessoa, Paraíba, Brazil.

^c^ Environmental Company of the State of São Paulo, Ribeirão Preto, São Paulo, Brazil.

^d^ National Institute for Agrarian and Veterinary Research (INIAV), I.P., 4485-655 Vila do Conde, Portugal.

*** Corresponding author:**

Prof. Eliana Guedes Stehling ([elianags@usp.br](mailto:elianags@usp.br))

Mailing address: Av. do Café, S/N, Monte Alegre, Ribeirão Preto, 14040-903, Brazil

Phone: + 55 16 3315 0285

**Supplementary Table S1.** Primers used for identification of *Enterococcus* species

| Target gene | Primer sequence (5'-3') | | Amplicon  size (bp) | AT (°C)^1^ | Reference |
| --- | --- | --- | --- | --- | --- |
|  | **Forward** | **Reverse** |  |  |  |
| *sodA*  (*Enterococcus* spp.) | ACAGTTGAAGAATTATTAGCAGACTTTT | GCTAGTTTACCGTCTTTAACG | 269 | 54 | Layton et. (2019) |
| *ddl*  (*E. faecium*) | AAAAAACAATAGAAGAATTAT | TGCTTTTTTGAATTCTTCTTTA | 215 | 55 | Dutka-malen et al. (1995) |
| *ddl*  (*E. faecalis*) | ACTTATGTGACTACTAACTTAACC | TAATGGTGAATCTTGGTTTGG | 360 | 55 | Dutka-malen et al. (1995) |
| 16S rRNA | AGAGTTTGATCCTGGCTCAG | ACGGCTACCTTGTTACGACTT | 1499 | 57 | Weisburg et al. (1991) |

^1^ Annealing temperature, AT.

**Supplementary Table S2.** Primers used for detection of antimicrobial resistance genes

| Target gene | Primer sequence (5'-3') | | Amplicon  size (bp) | AT (°C)^1^ | Reference |
| --- | --- | --- | --- | --- | --- |
|  | **Forward** | **Reverse** |  |  |  |
| *vanA* | AAATGTGCGAAAAACCT | TCCTGATGAATACGAAAGAT | 732 | 54 | Dutka-Malen et al. (1995) |
| *vanB* | ACCTACCCTGTCTTTGTGAA | AATGTCTGCTGGAACGATA | 300 | 54 | Zanella et al. (2003) |
| *erm*(A) | TCTAAAAAGCATGTAAAAGAA | CTTCGATAGTTTATTAATATTAGT | 645 | 47 | Sutcliffe et al. (1996) |
| *erm*(B) | GAAAAGGTACTCAACATA | AGTAACGGTACTTAAATTGTTTAC | 639 | 47 | Sutcliffe et al. (1996) |
| *erm*(C) | TCAAAACATAATATAGATAAA | GCTAATATTGTTTAAATCGTCAAT | 642 | 47 | Sutcliffe et al. (1996) |
| *mefAE* | AGTATCATTAATCACTAGTGC | TTCTTCTGGTACTAAAAGTGG | 348 | 47 | Sutcliffe et al. (1996) |
| *ant(4′)-Ia* | GGAAGCAGAGTTCAGCCATG | TGCCTGCATATTCAAACAGC | 266 | 55 | Kobayashi et al. (2001) |
| *aac(6′)-Ie-aph(2″)-Ia* | CCAAGAGCAATAAGGGCATA | CACTATCATACCACTACCG | 222 | 60 | Van de Klundert et al. (1993) |
| *aph(3′)-IIIa* | GCCGATGTGGATTGCGAAAA | GCTTGATCCCCAGTAAGTCA | 269 | 60 | Van de Klundert et al. (1993) |
| *ant(6′)-Ia* | ACTGGCTTAATCAATTTGGG | GCCTTTCCGCCACCTCACCG | 597 | 60 | Sepúlveda et al. (2017) |
| *aph(2")-Ib* | CTTGGACGCTGAGATATATGAGCAC | GTTTGTAGCAATTCAGAAACACCCTT | 867 | 55 | Vakulenko et al. (2003) |
| *aph(2")-Ic* | CCACAATGATAATGACTCAGTTCCC | CCACAGCTTCCGATAGCAAGAG | 444 | 55 | Vakulenko et al. (2003) |
| *aph(2")-Id* | GTGGTTTTTACAGGAATGCCATC | CCCTCTTCATACCAATCCATATAACC | 641 | 55 | Vakulenko et al. (2003) |
| *cfr* | TGAAGTATAAAGCAGGTTGGGAGTCA | ACCATATAATTGACCACAAGCAGC | 746 | 48 | Lee et al. (2017) |
| *cfr*(B) | TGAGCATATACGAGTAACCTCAAGA | CGCAAGCAGCGTCTATATCA | 293 | 58 | Yoo et al. (2020) |
| *cfr*(D) | AGAAGTCGCAACAAGTGAGGA | GCAACTGCATGAGTCAAAGAA | 595 | 60 | Ruiz-Ripa et al. (2020) |
| *poxtA* | GGTGGATTTACCGACACCGT | TCAATGCAGAGCAGGAAGCA | 791 | 51 | Yi et al. (2022) |
| *fexA* | GTACTTGTAGGTGCAATTACGGCTGA | CGCATCTGAGTAGGACATAGCGTC | 1272 | 58 | Kehrenberg et al. (2006) |
| *fexB* | TTCCCACTATTGGTGAAAGGAT | GCAATTCCCTTTTATGGACGTT | 816 | 55 | Liu et al. (2012) |
| *optrA* | TACTTGATGAACCTACTAACCA | CCTTGAACTACTGATTCTCGG | 422 | 55 | Brenciani et al. (2016) |
| *tet*(L) | TCGTTAGCGTGCTGTCATTC | GTATCCCACCAATGTAGCCG | 267 | 55 | Ng et al. (2021) |
| *tet*(M) | GTGGACAAAGGTACAACGAG | CGGTAAAGTTCGTCACACAC | 406 | 55 | Ng et al. (2021) |
| *tet*(O) | AACTTAGGCATTCTGGCTCAC | TCCCACTGTTCCATATCGTCA | 515 | 55 | Ng et al. (2021) |

^1^ Annealing temperature, AT.

**Supplementary Table S3.** Primers used for detection of virulence genes

| Target gene | Primer sequence (5'-3') | | Amplicon  size (bp) | AT  (°C)^1^ | Reference |
| --- | --- | --- | --- | --- | --- |
|  | **Forward** | **Reverse** |  |  |  |
| *ace* | AAGTAGAATTAGATCCACAC | TCTATCACATTCGGTTGCG | 320 | 50 | Mannu et al. (2003) |
| *cylA* | TGGATGATAGTGATAGGAAGT | TCTACAGTAAATCTTTCGTCA | 517 | 56 | Mannu et al. (2003) |
| *asa1* | CCAGTAATCAGTCCAGAAACAACC | TAGCTTTTTTCATTCTTGTGTTTGTT | 406 | 58 | Mannu et al. (2003) |
| *gelE* | ACCCCGTATCATTGGTTT | ACGCATTGCTTTTCCATC | 419 | 54 | Eaton et al. (2001) |
| *esp* | TTGCTAATGCTAGTCCACGACC | GCGTCAACACTTGCATTGCCGAA | 933 | 65 | Eaton et al. (2001) |
| *hyl* | ACAGAAGAGCTGCAGGAAATG | GACTGACGTCCAAGTTTCCAA | 276 | 56 | Vankerckhoven et al. (2004) |

^1^ Annealing temperature, AT.

**Supplementary Table S4.** Primers used for detection of plasmid replicons

| Target gene | Primer sequence (5'-3') | | Amplicon size (bp) | AT  (°C)^1^ | Reference |
| --- | --- | --- | --- | --- | --- |
|  | **Forward** | **Reverse** |  |  |  |
| *rep*_1_ | TCGCTCAATCACTACCAAGC | CTTGAACGAGTAAAGCCCTT | 624 | 56 | Jensen et al. (2010) |
| *rep*_2_ | GAGAACCATCAAGGCGAAAT | ACCAGAATAAGCACTACGTACAATCT | 630 | 56 | Jensen et al. (2010) |
| *rep*_3_ | CCTAATGTATATAATTTTGGTACATAT | ACATTTTCCTCAAAGAACAT | 402 | 52 | Jensen et al. (2010) |
| *rep*_4_ | ACTATGTCGTTGAGTCTAATGACT | AGCAAGATAGAATATTTACTTTTAAGTTT | 430 | 52 | Jensen et al. (2010) |
| *rep*_5_ | ATGTGTAATAAATTAAAAGAGCA | ATTGTCTTGATTTATCTATCTTG | 637 | 52 | Jensen et al. (2010) |
| *rep*_6_ | ACGAATGAAAGATAAAGGAGTAG | TAAATTCTAGTTTGGCAATCTTAT | 551 | 56 | Jensen et al. (2010) |
| *rep*_7_ | AGACGTAATATGCGTRTTGA | CCAAAATAYTTYGTTTCTGG | 227 | 56 | Jensen et al. (2010) |
| *rep*_8_ | TAGATACGACAAAAGAAGAATTACA | CCAATCATGTAATGTTACAACC | 394 | 56 | Jensen et al. (2010) |
| *rep*_9_ | GCTCGATCARTTTTCAGAAG | CGCAAACATTTGTCWATTTCTT | 201 | 56 | Jensen et al. (2010) |
| *rep*_10_ | TATAAAGGCTCTCAGAGGCT | CCAAATTCGAGTAAGAGGTA | 382 | 56 | Jensen et al. (2010) |
| *rep*_11_ | TCTAGAATGCGTAAAAAGG | CCTTTGAAGATWGCRGTWAG | 500 | 52 | Jensen et al. (2010) |
| *rep*_12_ | GAGCCTATAACAGAGTACACA | CAAATATAGGCTTTGTAGTTC | 470 | 52 | Jensen et al. (2010) |
| *rep*_13_ | ATGATGCAATATATTAAGCA | TACCAGAATAYTTAGCCATTTC | 402 | 52 | Jensen et al. (2010) |
| *rep*_14_ | GAAAGYTTRGATAGYTTTGC | RTTTTGRCTTTCTTSYTTCA | 164 | 52 | Jensen et al. (2010) |
| *rep*_15_ | CAGTAGAAGAAAATTATAAAGAAC | GTTATGGCTGGTTTTAATAAA | 327 | 52 | Jensen et al. (2010) |
| *rep*_16_ | CAGGAAAACACTTCGTTTAT | CTTCTATATCACTATCATTGTCATT | 592 | 52 | Jensen et al. (2010) |
| *rep*_17_ | TACTAACTGTTGGTAATTCGTTAAAT | ATCAAGGACTCAACCGTAATT | 604 | 52 | Jensen et al. (2010) |
| *rep*_18_ | ACACCAGTCGAAATGAATTT | AGGAATATCAAGTAATTCATGAAAGT | 462 | 56 | Jensen et al. (2010) |
| *rep*_19_ | GWGATCGCTTARAYTTATCTAT | YMTTGTTSTGGMAATTCTT | 543 | 52 | Jensen et al. (2010) |
| *rep*_Unique_ | GTATTAACACACTGGACTC | TCAGTGTAGGCAATAACCC | 199 | 52 | Jensen et al. (2010) |

^1^ Annealing temperature, AT.

**Supplementary Table S5.** Primers used for MLST analysis

| Target gene | Species^1^ | Primer sequence (5'-3') | | Amplicon  size (bp) | AT  (°C)^2^ | Reference |
| --- | --- | --- | --- | --- | --- | --- |
|  |  | **Forward** | **Reverse** |  |  |  |
| *gdh* | Efc | GGCGCACTAAAAGATATGGT | CCAAGATTGGGCAACTTCGTCCCA | 530 | 52 | Ruiz-Garbajosa et al. (2006) |
| *gyd* | Efc | CAAACTGCTTAGCTCCAATGGC | CATTTCGTTGTCATACCAAGC | 395 | 52 | Ruiz-Garbajosa et al. (2006) |
| *pstS* | Efc | CGGAACAGGACTTTCGC | ATTTACATCACGTTCTACTTGC | 583 | 52 | Ruiz-Garbajosa et al. (2006) |
| *gki* | Efc | GATTTTGTGGGAATTGGTATGG | ACCATTAAAGCAAAATGATCGC | 438 | 52 | Ruiz-Garbajosa et al. (2006) |
| *aroE* | Efc | TGGAAAACTTTACGGAGACAGC | GTCCTGTCCATTGTTCAAAAGC | 459 | 52 | Ruiz-Garbajosa et al. (2006) |
| *xpt* | Efc | AAAATGATGGCCGTGTATTAGG | AACGTCACCGTTCCTTCACTTA | 456 | 52 | Ruiz-Garbajosa et al. (2006) |
| *yqiL* | Efc | CAGCTTAAGTCAAGTAAGTGCCG | GAATATCCCTTCTGCTTGTGCT | 436 | 52 | Ruiz-Garbajosa et al. (2006) |
| *adk* | Efm | TATGAACCTCATTTTAATGGG | GTTGACTGCCAAACGATTTT | 437 | 50 | Homan et al. (2002) |
| *atpA* | Efm | CGGTTCATACGGAATGGCACA | AAGTTCACGATAAGCCACGG | 556 | 50 | Homan et al. (2002) |
| *ddl* | Efm | GAGACATTGAATATGCCTTATG | AAAAAGAAATCGCACCG | 465 | 50 | Homan et al. (2002) |
| *gdh* | Efm | GGCGCACTAAAAGATATGGT | CCAAGATTGGGCAACTTCGTCCCA | 530 | 50 | Homan et al. (2002) |
| *gyd* | Efm | CAAACTGCTTAGCTCCAAGGC | CATTTCGTTGTCATACCAAGC | 395 | 50 | Homan et al. (2002) |
| *purK* | Efm | GCAGATTGGCACATTGAAAGT | TACATAAATCCCCCTGTTTY | 492 | 50 | Homan et al. (2002) |
| *pstS* | Efm | TTGAGCCAAGTCGAAGCTGGAG | CGTGATCACGTTCTACTTCC | 583 | 50 | Homan et al. (2002) |

^1^ *E. faecalis*, Efc; *E. faecium*, Efm. ^2^ Annealing temperature, AT.

**Supplementary Table S6.** Bacterial isolates carrying the *optrA* gene (n=67) according to species, isolation source, city, and collection date

| **Isolate** | **Species^1^** | **Isolation source** | **City (state of São Paulo)** | **Collection date** |
| --- | --- | --- | --- | --- |
| EW1472 | Efc | Cachoeirinha Stream | Olímpia | July, 2021 |
| EW1474 | Efc | Onça Stream | Palmares Paulista | July, 2021 |
| EW1477 | Efc | Matadouro Stream | Olímpia | July, 2021 |
| EW1478 | Efc | Olhos d'água Stream | Olímpia | July, 2021 |
| EW1531 | Efc | Turvo River | Guapiaçu | July, 2021 |
| EW1572 | Efc | Bagres Stream | Batatais | October, 2021 |
| EW1577 | Efc | Sapucaí River | São Jose da Bela Vista | October, 2021 |
| EW1578 | Efc | Carmo River | Ituverava | October, 2021 |
| EW1579 | Efc | Carmo River | Ituverava | October, 2021 |
| EW1581 | Efc | Pinheirinho Stream | Santo Antônio da Alegria | October, 2021 |
| EW1585 | Efc | Jaguara Stream | Rifaina | October, 2021 |
| EW1592 | Efc | Bagres Stream | Franca | October, 2021 |
| EW1593 | Efc | Roque River | Pirassununga | October, 2021 |
| EW1595 | Efc | Itupeva River | Pirassununga | October, 2021 |
| EW1596 | Efc | Itupeva River | Pirassununga | October, 2021 |
| EW1598 | Efc | Mogi-Guaçu River | Santa Rita do Passa Quatro | October, 2021 |
| EW1603 | Efc | Água Branca Stream | Itirapina | October, 2021 |
| EW1604 | Efc | Água Branca Stream | Itirapina | October, 2021 |
| EW1605 | Efc | Pardo River | Guaíra | October, 2021 |
| EW1606 | Efc | Pardo River | Guaíra | October, 2021 |
| EW1607 | Efc | Mandembo Stream | Bebedouro | October, 2021 |
| EW1608 | Efc | Mandembo Stream | Bebedouro | October, 2021 |
| EW1612 | Efc | Ribeirão Preto Stream | Ribeirão Preto | November, 2021 |
| EW1613 | Efc | Ribeirão Preto Stream | Ribeirão Preto | November, 2021 |
| EW1614 | Efc | Piedade Stream | São José do Rio Preto | November, 2021 |
| EW1617 | Efc | Preto River | Ipiguá | November, 2021 |
| EW1618 | Efc | Preto River | Ipiguá | November, 2021 |
| EW1624 | Efc | Jacaré-Guaçu River | Ibitinga | November, 2021 |
| EW1625 | Efc | Jacaré-Guaçu River | Ibitinga | November, 2021 |
| EW1626 | Efc | Monjolinho River | São Carlos | November, 2021 |
| EW1627 | Efc | Monjolinho River | São Carlos | November, 2021 |
| EW1628 | Efc | Pardo River | Ribeirão Preto | November, 2021 |
| EW1629 | Efc | Pardo River | Ribeirão Preto | November, 2021 |
| EW1630 | Efc | Sertãozinho Stream | Sertãozinho | November, 2021 |
| EW1631 | Efc | Sertãozinho Stream | Sertãozinho | November, 2021 |
| EW1633 | Efc | Mogi-Guaçu River | Pitangueiras | November, 2021 |
| EW1635 | Efc | Onças River | Luís Antônio | November, 2021 |
| EW1636 | Efc | Billuca Stream | São José do Rio Preto | November, 2021 |
| EW1642 | Efc | Turvo River | Paraíso | November, 2021 |
| EW1647 | Efc | São Domingos Stream | Catiguá | November, 2021 |
| EW1648 | Efc | São Domingos Stream | Catiguá | November, 2021 |
| EW1649 | Efc | São Domingos Stream | Catanduva | November, 2021 |
| EW1650 | Efc | São Domingos Stream | Catanduva | November, 2021 |
| EW1651 | Efc | Jacaré-Pepira River | Dourado | November, 2021 |
| EW1652 | Efc | Cachoeirinha River | Olímpia | November, 2021 |
| EW1660 | Efc | Ribeirão Marinheiro | Pedranópolis | November, 2021 |
| EW1662 | Efc | Grande River | Ouroeste | November, 2021 |
| EW1667 | Efc | Preto River | Palestina | November, 2021 |
| EW1668 | Efc | Rico Stream | Jaboticabal | November, 2021 |
| EW1670 | Efc | Mogi-Guaçu River | Pirassununga | November, 2021 |
| EW1672 | Efc | Mogi-Guaçu River | Porto Ferreira | November, 2021 |
| EW1706 | Efc | Cachoeirinha River | Olímpia | January, 2022 |
| EW1469 | Efm | São Domingos Stream | Catanduva | July, 2021 |
| EW1475 | Efm | Biluca Stream | São José do Rio Preto | July, 2021 |
| EW1479 | Efm | Matadouro Stream | Olímpia | July, 2021 |
| EW1483 | Efm | São José dos Dourados River | Monte Aprazível | July, 2021 |
| EW1587 | Efm | Sapucaí River | São José da Barra | October, 2021 |
| EW1597 | Efm | Mogi-Guaçu River | Santa Rita do Passa Quatro | October, 2021 |
| EW1616 | Efm | Olhos d'água Stream | Olímpia | November, 2021 |
| EW1637 | Efm | Turvo River | Guapiaçu | November, 2021 |
| EW1638 | Efm | Cachoeirinha River | Olímpia | November, 2021 |
| EW1639 | Efm | Cachoeirinha River | Olímpia | November, 2021 |
| EW1641 | Efm | Matadouro Stream | Olímpia | November, 2021 |
| EW1643 | Efm | Cachoeirinha River | Olímpia | November, 2021 |
| EW1681 | Efm | Jacaré-Guaçu River | Araraquara | December, 2021 |
| EW1682 | Efm | Jacaré-Guaçu River | Ribeirão Bonito | December, 2021 |
| EW1691 | Efm | Itaqueri River | Itirapina | December, 2021 |

^1^ *E. faecalis*, Efc; *E. faecium*, Efm.

**Supplementary Table S7.** Antimicrobial susceptibility of *optrA*-positive *E. faecalis* and *E. faecium* isolates (n=67)

| **Strain^1^** | **Species^2^** | **Antimicrobial resistance profile^3^** | **MIC (mg/L)^4^** | | **HLAR^5^** | | **HLCR^6^** |
| --- | --- | --- | --- | --- | --- | --- | --- |
|  |  |  | **LNZ** | **VAN** | **GEN** | **STP** | **CIP** |
| EW1472 | Efc | LNZ, IMP, CIP, LVX, NOR, TET, DOX, MIN | 8 | 2 | + | + | - |
| EW1474 | Efc | LNZ, TEC, IMP, CIP, LVX, NOR, TET, DOX, MIN, NIT | 8 | 1 | + | + | - |
| EW1477 | Efc | LNZ, TEC, IMP, CIP, LVX, NOR, TET, DOX, MIN, NIT | 128 | 2 | + | + | - |
| EW1478 | Efc | LNZ, TEC, IMP | 8 | 1 | - | - | - |
| EW1531 | Efc | LNZ, TEC, IMP, CIP, LVX, NOR, TET, DOX, MIN | 8 | 2 | - | - | - |
| EW1572 | Efc | LNZ, TEC, IMP, CIP, TET, DOX, MIN | 16 | 2 | - | - | - |
| EW1577 | Efc | LNZ, TEC, IMP, CIP, LVX, NOR, TET | 16 | 2 | + | + | - |
| EW1578 | Efc | LNZ, TEC, IMP, CIP, LVX, NOR, TET, DOX, MIN | 16 | 2 | + | + | - |
| EW1579 | Efc | LNZ, IMP, CIP, LVX, NOR, TET, DOX, MIN, CHL | 8 | 2 | + | + | - |
| EW1581 | Efc | LNZ, IMP, TET, DOX, MIN | 16 | 2 | - | - | - |
| EW1585 | Efc | LNZ, TEC, IMP, CIP, LVX, NOR, TET, DOX, MIN | 16 | 2 | - | - | - |
| EW1592 | Efc | IMP, TET, DOX, MIN, CIP | 0.5 | 2 | + | - | - |
| EW1593 | Efc | LNZ, TEC, IMP, CIP, LVX, NOR, TET, DOX, MIN, NIT | 8 | 2 | + | + | + |
| EW1595 | Efc | LNZ, TEC, IMP, TET, DOX, MIN, CHL | 16 | 2 | + | - | + |
| EW1596 | Efc | AMP, LNZ, TEC, IMP, CIP, LVX, NOR, TET, DOX, MIN, NIT | 16 | 2 | - | - | - |
| EW1598 | Efc | LNZ, TEC, IMP, CIP, LVX, NOR, TET, DOX, MIN, NIT, CHL | 16 | 2 | + | + | + |
| EW1603 | Efc | LNZ, TEC, IMP, TET, DOX, MIN, CHL | 16 | 2 | + | - | + |
| EW1604 | Efc | LNZ, TEC, IMP, CIP, LVX, NOR, TET, DOX, MIN, CHL | 16 | 2 | - | - | - |
| EW1605 | Efc | LNZ, TEC, IMP, CIP, LVX, NOR, TET, DOX, MIN, CHL | 16 | 2 | + | + | - |
| EW1606 | Efc | LNZ, IMP, CIP, LVX, NOR, TET, DOX, MIN, CHL, RIF | 16 | 2 | + | + | - |
| EW1607 | Efc | LNZ, TEC, IMP, TET, DOX, MIN, CHL, FOS, RIF | 16 | 1 | - | - | - |
| EW1608 | Efc | LNZ, TEC, IMP, TET, DOX, MIN, CHL | 16 | 1 | - | - | - |
| EW1612 | Efc | LNZ, TEC, IMP, CIP, LVX, NOR, TET, DOX, MIN, CHL | 16 | 1 | - | - | - |
| EW1613 | Efc | LNZ, IMP, TET, DOX, MIN, CHL | 32 | 1 | + | + | - |
| EW1614 | Efc | LNZ, TEC, IMP, TET, DOX, MIN, CHL | 8 | 1 | - | - | - |
| EW1617 | Efc | LNZ, TEC, IMP, CIP, LVX, NOR, TET, DOX, MIN, CHL | 8 | 1 | + | + | - |
| EW1618 | Efc | LNZ, TEC, IMP, TET, DOX, MIN, CHL | 16 | 1 | - | - | - |
| EW1624 | Efc | LNZ, IMP, CIP, LVX, NOR, TET, DOX, MIN, CHL | 8 | 1 | - | - | - |
| EW1625 | Efc | LNZ, TEC, IMP, CIP, LVX, NOR, TET, DOX, MIN, CHL | 16 | 1 | - | - | - |
| EW1626 | Efc | LNZ, TEC, IMP, CIP, LVX, NOR, TET, DOX, MIN, CHL, RIF | 16 | 4 | + | + | - |
| EW1627 | Efc | LNZ, IMP, CIP, LVX, NOR, TET, DOX, MIN, NIT, CHL | 16 | 4 | + | - | - |
| EW1628 | Efc | LNZ, TEC, IMP, TET, DOX, MIN, CHL | 16 | 2 | + | - | - |
| EW1629 | Efc | LNZ, IMP, CIP, LVX, NOR, TET, DOX, MIN, CHL | 16 | 2 | + | + | + |
| EW1630 | Efc | LNZ, IMP, CIP, LVX, NOR, TET, DOX, MIN, CHL | 16 | 4 | + | + | - |
| EW1631 | Efc | LNZ, TEC, IMP, CIP, LVX, NOR, TET, DOX, MIN, CHL | 16 | 2 | + | - | + |
| EW1633 | Efc | LNZ, TEC, IMP, TET, DOX, MIN, CHL | 32 | 2 | + | - | - |
| EW1635 | Efc | IMP, CIP, LVX, NOR, TET, DOX, MIN, CHL | 1 | 2 | - | - | - |
| EW1636 | Efc | LNZ, TEC, IMP, TET, DOX, MIN, CHL | 128 | 2 | + | + | - |
| EW1642 | Efc | LNZ, IMP, TET, DOX, MIN, CHL | 16 | 4 | - | + | - |
| EW1647 | Efc | LNZ, TEC, IMP, CIP, LVX, NOR, TET, DOX, MIN, CHL, FOS, RIF | 16 | 2 | + | - | - |
| EW1648 | Efc | LNZ, TEC, IMP, CIP, LVX, NOR, TET, DOX, MIN, CHL | 16 | 4 | - | - | - |
| EW1649 | Efc | LNZ, TEC, IMP, TET, DOX, MIN, CHL | 16 | 4 | - | - | - |
| EW1650 | Efc | LNZ, TEC, IMP, CIP, LVX, NOR, TET, DOX, MIN, CHL | 8 | 2 | + | + | - |
| EW1651 | Efc | LNZ, TEC, IMP, CIP, LVX, NOR, TET, DOX, MIN, CHL | 16 | 4 | + | + | - |
| EW1652 | Efc | AMP, LNZ, VAN, IMP | 16 | 8 | - | - | - |
| EW1660 | Efc | LNZ, TEC, IMP, CIP, LVX, NOR, TET, DOX, MIN, CHL | 8 | 2 | + | + | - |
| EW1662 | Efc | LNZ, VAN, TEC, IMP, CIP, LVX, NOR, CHL | 128 | 8 | - | - | - |
| EW1667 | Efc | IMP, NIT, CIP, TET | 0.5 | 4 | - | - | - |
| EW1668 | Efc | LNZ, TEC, IMP, TET, DOX, MIN, CHL | 128 | 4 | - | + | - |
| EW1670 | Efc | LNZ, TEC, IMP, CIP, LVX, NOR, TET, DOX, MIN, NIT, CHL | 128 | 4 | - | - | - |
| EW1672 | Efc | LNZ, IMP, TET, DOX, MIN, NIT, CHL | 128 | 4 | - | - | - |
| EW1706 | Efc | LNZ, TEC, IMP, NIT | 16 | 4 | - | - | - |
| EW1469 | Efm | AMP, IMP, CIP, LVX, NOR, TET, DOX, MIN, NIT, ERI, FOS, RIF | 2 | 2 | - | - | - |
| EW1475 | Efm | LNZ, TEI, IMP, NIT, ERI, FOS, RIF | 8 | 4 | - | - | - |
| EW1479 | Efm | LNZ, IMP, CIP, LVX, NOR, NIT, ERI | 8 | 2 | - | - | - |
| EW1483 | Efm | LNZ, IMP, NIT, ERI, RIF | 8 | 2 | - | - | - |
| EW1587 | Efm | LNZ, IMP, TEI, TET, DOX, MIN, CIP, LRV, NOR, FOS, CHL | 128 | 2 | - | - | - |
| EW1597 | Efm | LNZ, IMP, CIP, LVX, NOR, TET, DOX, MIN, NIT, CHL, ERI, FOS, RIF | 8 | 2 | + | + | - |
| EW1616 | Efm | LNZ, IMP, NIT, RIF | 8 | 2 | - | - | - |
| EW1637 | Efm | LNZ, IMP, NIT, FOS, RIF | 8 | 2 | - | - | - |
| EW1638 | Efm | LNZ, IMP, NIT, FOS, RIF | 8 | 2 | - | - | - |
| EW1639 | Efm | LNZ, TEI, IMP, CIP, LVX, NOR, NIT, ERI, FOS, RIF | 16 | 1 | - | - | - |
| EW1641 | Efm | AMP, LNZ, TEI, IMP, NIT, ERI, FOS, RIF | 8 | 1 | - | - | - |
| EW1643 | Efm | LNZ, IMP, ERI, FOS, RIF | 8 | 1 | - | - | - |
| EW1681 | Efm | LNZ, IMP, CIP, LVX, NOR, CHL, ERI, FOS, RIF | 8 | 1 | - | - | - |
| EW1682 | Efm | LNZ, TEI, IMP, TET, DOX, MIN, NIT, CHL, ERI, FOS, RIF | 128 | 1 | - | - | - |
| EW1691 | Efm | LNZ, IMP, TET, DOX, MIN, NIT, ERI, RIF | 8 | 1 | + | + | - |

^1^ Isolates obtained from environmental samples in this study. ^2^ *E. faecalis*, Efc; *E. faecium*, Efm. ^3^ Ciprofloxacin, CIP; Levofloxacin, LVX; Norfloxacin, NOR; Erythromycin, ERI; Rifampicin, RIP; Linezolid, LNZ; Tetracycline, TET; Doxycycline, DOX; Minocycline, MIN; Nitrofurantoin, NIT; Vancomycin, VAN; Fosfomycin, FOS; Chloramphenicol, CHL. ^4^ Minimal inhibitory concentration, MIC. Breakpoints according to CLSI, 2020 [M100, 30th ed.]. ^5^ HLGR: High-level gentamicin resistance; HLSR: High-level streptomycin resistance. ^6^ HLCR: High-level ciprofloxacin resistance.

**Supplementary Table S8.** Mutations in the 23S rRNA of *optrA*-positive *E. faecalis* and *E. faecium* isolates (n=67)

| Strain | Species^1^ | Mutation^2^ | LNZ (MIC mg/L)^3^ |
| --- | --- | --- | --- |
| EW1472 | Efc | G2156A, G2158A, A2571G | 8 |
| EW1474 | Efc | ND | 8 |
| EW1477 | Efc | A2571G | 128 |
| EW1478 | Efc | A2130T, T2131A, G2134A, C2136T, G2153A, A2154T, T2179C, T2182C, A2189G, A2190C, C2192A, A2210G, T2356A | 8 |
| EW1531 | Efc | ND | 8 |
| EW1572 | Efc | ND | 16 |
| EW1577 | Efc | ND | 16 |
| EW1578 | Efc | ND | 16 |
| EW1579 | Efc | ND | 8 |
| EW1581 | Efc | ND | 16 |
| EW1585 | Efc | ND | 16 |
| EW1592 | Efc | T2131A, G2134A, C2136T, G2153A, A2154T, T2179C, T2182C, A2189G, A2190C, C2192A, A2210G, T2356A | 0.5 |
| EW1593 | Efc | ND | 8 |
| EW1595 | Efc | ND | 16 |
| EW1596 | Efc | ND | 16 |
| EW1598 | Efc | ND | 16 |
| EW1603 | Efc | ND | 16 |
| EW1604 | Efc | ND | 16 |
| EW1605 | Efc | ND | 16 |
| EW1606 | Efc | C2162T | 16 |
| EW1607 | Efc | ND | 16 |
| EW1608 | Efc | ND | 16 |
| EW1612 | Efc | T2148C | 16 |
| EW1613 | Efc | ND | 32 |
| EW1614 | Efc | ND | 8 |
| EW1617 | Efc | ND | 8 |
| EW1618 | Efc | ND | 16 |
| EW1624 | Efc | ND | 8 |
| EW1625 | Efc | ND | 16 |
| EW1626 | Efc | C2162T | 16 |
| EW1627 | Efc | C2162T | 16 |
| EW1628 | Efc | ND | 16 |
| EW1629 | Efc | ND | 16 |
| EW1630 | Efc | ND | 16 |
| EW1631 | Efc | ND | 16 |
| EW1633 | Efc | ND | 32 |
| EW1635 | Efc | ND | 1 |
| EW1636 | Efc | ND | 128 |
| EW1642 | Efc | ND | 16 |
| EW1647 | Efc | ND | 16 |
| EW1648 | Efc | ND | 16 |
| EW1649 | Efc | ND | 16 |
| EW1650 | Efc | ND | 8 |
| EW1651 | Efc | ND | 16 |
| EW1652 | Efc | ND | 16 |
| EW1660 | Efc | ND | 8 |
| EW1662 | Efc | ND | 128 |
| EW1667 | Efc | A2130T, T2131A, G2134A, C2136T, G2153A, A2154T, T2179C, T2182C, A2189G, A2190C, C2192A, A2210G, T2356A | 0.5 |
| EW1668 | Efc | ND | 128 |
| EW1670 | Efc | ND | 128 |
| EW1672 | Efc | A2130T, T2131A, G2134A, C2136T, G2153A, A2154T, T2179C, T2182C, A2189G, A2190C, C2192A, A2210G, T2356A | 128 |
| EW1706 | Efc | A2130T, T2131A, G2134A, C2136T, G2153A, A2154T, T2179C, T2182C, A2189G, A2190C, C2192A, A2210G, T2356A | 16 |
| EW1469 | Efm | A2210G | 2 |
| EW1475 | Efm | A2134G, A2140G, T2146C, G2162A, C2163A, C2180T, C2206G, A2208C, A2211T, T2216G, G2218C, C2355G, A2357T, G2362C, T2699C, C2700T, T2701C, A2706G, G2707A, T2713C | 8 |
| EW1479 | Efm | ND | 8 |
| EW1483 | Efm | A2211G | 8 |
| EW1587 | Efm | G2595C, G2694A | 128 |
| EW1597 | Efm | ND | 8 |
| EW1616 | Efm | ND | 8 |
| EW1637 | Efm | ND | 8 |
| EW1638 | Efm | ND | 8 |
| EW1639 | Efm | ND | 16 |
| EW1641 | Efm | A2211G | 8 |
| EW1643 | Efm | A2211G | 8 |
| EW1681 | Efm | ND | 8 |
| EW1682 | Efm | ND | 128 |
| EW1691 | Efm | G2730A | 8 |

^1^ *E. faecalis*, Efc; *E. faecium*, Efm. ^2^ Not detected, ND. ^3^ Linezolid, LNZ.

**Supplementary Table S9.** MLST analysis of *E. faecalis* and *E. faecium* isolates co-harboring the *optrA* and *poxtA* genes (n=10)

| *Enterococcus faecalis* | | | | | | | | |
| --- | --- | --- | --- | --- | --- | --- | --- | --- |
| Isolate | *gdh* | *gyd* | *pstS* | *gki* | *aroE* | *xpt* | *yql* | ST^1^ |
| EW1636 | 1 | 7 | 11 | 11 | 3 | 4 | 2 | 283 |
| EW1662 | 14 | 6 | 7 | 36 | 13 | 35 | 28 | 1230 |
| EW1668 | 15 | 1 | 37 | 19 | 3 | 15 | 11 | 253 |
| EW1670 | 47 | 2 | 17 | 40 | 28 | 4 | 1 | 234 |
| EW1672 | 40 | 33 | 17 | 6 | 29 | 4 | 1 | 2126^#^ |
| *Enterococcus faecium* | | | | | | | | |
| Isolate | *atpA* | *ddl* | *gdh* | *purK* | *gyd* | *pstS* | *adk* | ST^1^ |
| EW1637 | 95 | 13 | 9 | 17 | 10 | 19 | 6 | 1221 |
| EW1638 | 25 | 13 | 9 | 17 | 10 | 26 | 6 | 3018^#^ |
| EW1639 | 38 | 1 | 1 | 2 | 1 | 252 | 1 | 3022^#^ |
| EW1681 | 34 | 9 | 1 | 6 | 1 | 58 | 1 | 3026^#^ |
| EW1682 | 38 | 89 | 6 | 2 | 6 | 252 | 1 | 3027^#^ |

^1^ Sequence type, ST. The symbol (^#^) represents isolates that have exhibited new sequence types due to new combinations of alleles. These new sequence types were assigned by the *Enterococcus* *faecalis* (<https://pubmlst.org/organisms/enterococcus-faecalis>) and *Enterococcus* *faecium* (<https://pubmlst.org/organisms/enterococcus-faecium>) databases from PubMLST (<https://pubmlst.org/>).


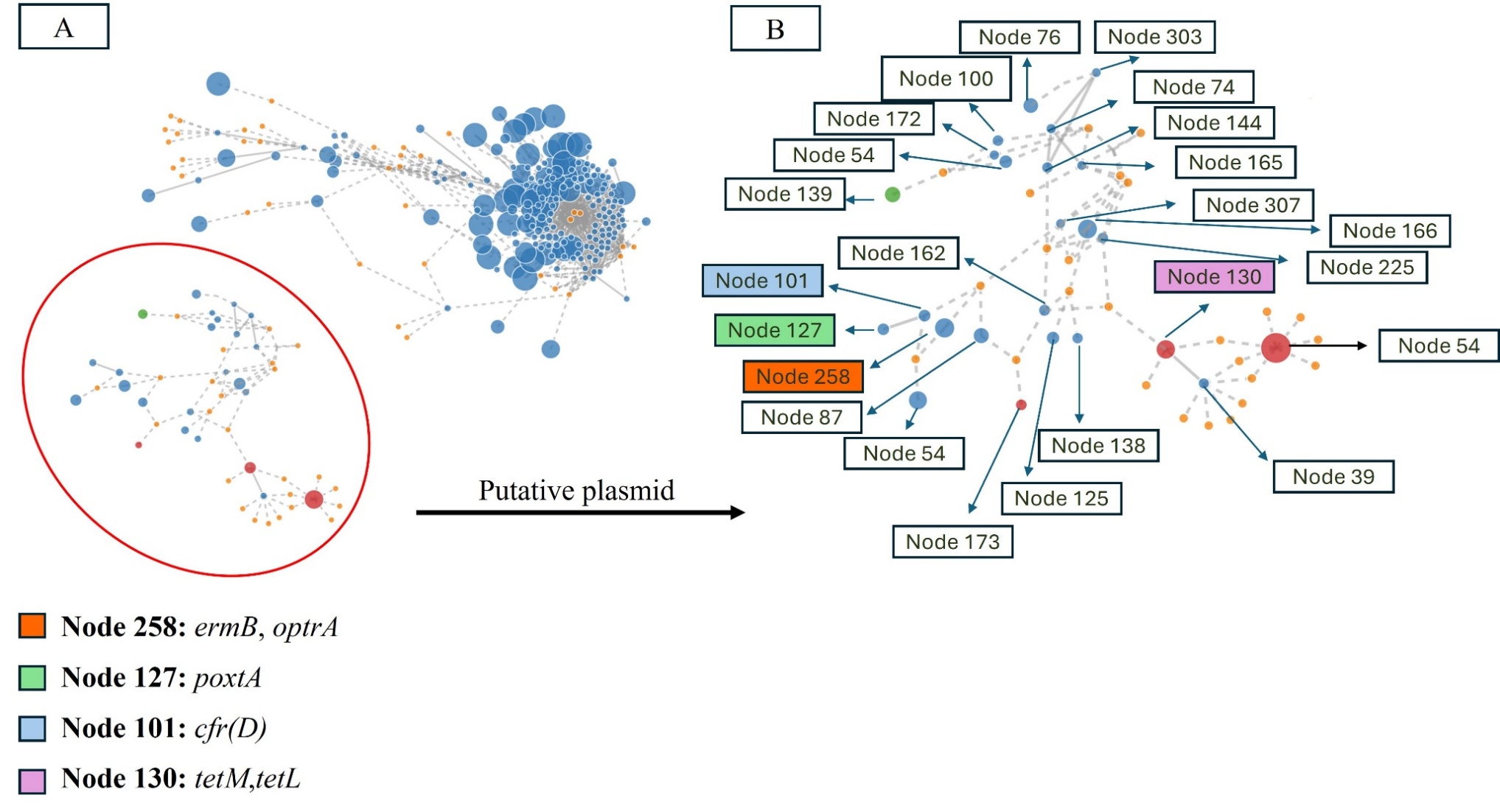
 **Supplementary Fig. S1.** PLACNETw illustrates the separation between putative plasmid and chromosome contigs. A) The region highlighted in red likely corresponds to plasmid-related contigs, as they are located near the replication origin and relaxase encoding gene. B) A detailed view of the plasmid cluster shown in Figure A, including node and gene annotations and supporting the potential association of these genes within the same plasmid.


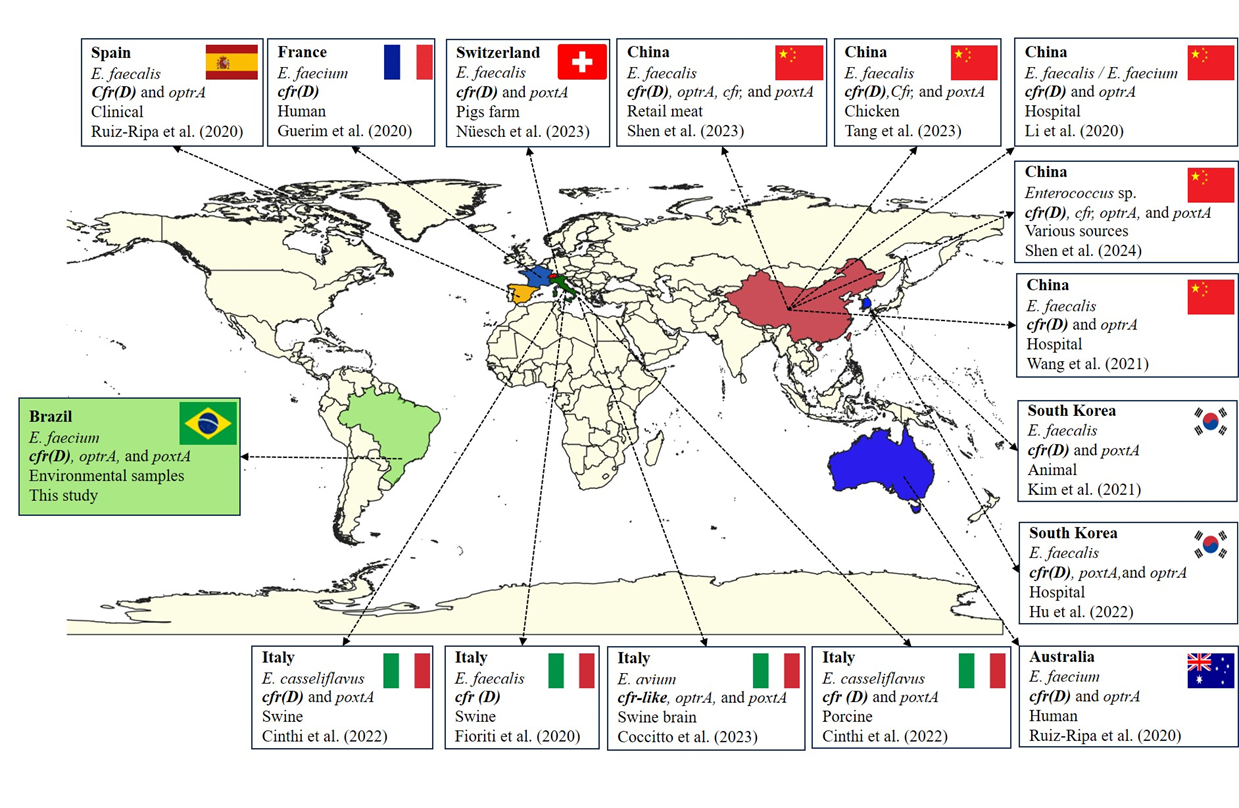


**Supplementary Fig. S2.** Prevalence and detection of the *cfr*(D) gene in *Enterococcus* isolates obtained worldwide.

**References**

Brenciani A, Morroni G, Vincenzi C, Manso E, Mingoia M, Giovanetti E, Varaldo PE (2016) Detection in Italy of two clinical *Enterococcus faecium* isolates carrying both the oxazolidinone and phenicol resistance gene *optrA* and a silent multiresistance gene *cfr* : Table 1. J. Antimicrob. Chemother. 71, 1118–1119. <https://doi.org/10.1093/jac/dkv438>.

Dutka-Malen S, Evers S, Courvalin P (1995) Detection of glycopeptide resistance genotypes and identification to the species level of clinically relevant enterococci by PCR. J Clin Microbiol 33, 24–27. <https://doi.org/10.1128/jcm.33.1.24-27.1995>.

Eaton TJ, Gasson MJ (2001) Molecular Screening of *Enterococcus* Virulence Determinants and Potential for Genetic Exchange between Food and Medical Isolates. Appl Environ Microbiol 67, 1628–1635. <https://doi.org/10.1128/AEM.67.4.1628-1635.2001>.

Homan WL, Tribe D, Poznanski S, Li M, Hogg G, Spalburg E, Van Embden JDA, Willems RJL (2002) Multilocus Sequence Typing Scheme for *Enterococcus faecium*. J Clin Microbiol 40, 1963–1971. <https://doi.org/10.1128/JCM.40.6.1963-1971.2002>.

Jensen LB, Garcia-Migura L, Valenzuela AJS, Løhr M, Hasman H, Aarestrup FM (2010) A classification system for plasmids from enterococci and other Gram-positive bacteria. J Microbiol Methods 80, 25–43. <https://doi.org/10.1016/j.mimet.2009.10.012>.

Kehrenberg C, Schwarz S (2006) Distribution of Florfenicol Resistance Genes *fexA* and *cfr* among Chloramphenicol-Resistant *Staphylococcus* Isolates. Antimicrob Agents Chemother 50, 1156–1163. <https://doi.org/10.1128/AAC.50.4.1156-1163.2006>.

Kobayashi N, Mahbub Alam Md, Nishimoto Y, Urasawa S, Uehara N, Watanabe N (2001) Distribution of aminoglycoside resistance genes in recent clinical isolates of *Enterococcus faecalis* , *Enterococcus faecium* and *Enterococcus avium*. Epidemiol. Infect. 126, 197–204. <https://doi.org/10.1017/S0950268801005271>.

Layton BA, Walters SP, Lam LH, Boehm AB (2010) *Enterococcus* species distribution among human and animal hosts using multiplex PCR. J Appl Microbiol 109, 539–547. <https://doi.org/10.1111/j.1365-2672.2010.04675.x>.

Lee SM, Huh HJ, Song DJ, Shim HJ, Park KS, Kang CI, Ki CS, Lee NY (2017) Resistance mechanisms of linezolid-nonsusceptible enterococci in Korea: low rate of 23S rRNA mutations in *Enterococcus faecium*. J Med Microbiol 66, 1730–1735. <https://doi.org/10.1099/jmm.0.000637>.

Liu H, Wang Y, Wu C, Schwarz S, Shen Z, Jeon B, Ding S, Zhang Q, Shen J (2012) A novel phenicol exporter gene, *fexB*, found in enterococci of animal origin. J Antimicrob Chemother 67, 322–325. <https://doi.org/10.1093/jac/dkr481>.

Mannu L, Paba A, Daga E, Comunian R, Zanetti S, Duprè I, Sechi LA (2003) Comparison of the incidence of virulence determinants and antibiotic resistance between *Enterococcus faecium* strains of dairy, animal and clinical origin. Int. J. Food Microbiol 88, 291–304. <https://doi.org/10.1016/S0168-1605(03)00191-0>.

Ng LK, Martin I, Alfa M, Mulvey M (2001) Multiplex PCR for the detection of tetracycline resistant genes. Molecular and Cellular Probes 15, 209–215. <https://doi.org/10.1006/mcpr.2001.0363>.

Ruiz-Garbajosa P, Bonten MJM, Robinson DA, Top J, Nallapareddy SR, Torres C, Coque TM, Cantón R, Baquero F, Murray BE, Del Campo R, Willems RJL (2006) Multilocus Sequence Typing Scheme for *Enterococcus faecalis* Reveals Hospital-Adapted Genetic Complexes in a Background of High Rates of Recombination. J Clin Microbiol 44, 2220–2228. <https://doi.org/10.1128/JCM.02596-05>.

Ruiz-Ripa L, Feßler AT, Hanke D, Eichhorn I, Azcona-Gutiérrez JM, Pérez-Moreno MO, Seral C, Aspiroz C, Alonso CA, Torres L, Alós JI, Schwarz S, Torres C (2020) Mechanisms of Linezolid Resistance Among Enterococci of Clinical Origin in Spain—Detection of *optrA*- and *cfr(D)-*Carrying *E. faecalis*. Microorganisms 8, 1155. <https://doi.org/10.3390/microorganisms8081155>.

Sepúlveda AM, Bello TH, Domínguez YM, Mella MS, Zemelman ZR, González RG (2007) Identificación molecular de enzimas modificantes de aminoglucósidos en cepas de Enterococcus spp. aisladas en hospitales de la Octava Región de Chile. Rev. méd. Chile 135. <https://doi.org/10.4067/S0034-98872007000500003>.

Sutcliffe J, Grebe T, Tait-Kamradt A, Wondrack L (1996) Detection of erythromycin-resistant determinants by PCR. Antimicrob Agents Chemother 40, 2562–2566. <https://doi.org/10.1128/AAC.40.11.2562>.

Vakulenko SB, Donabedian SM, Voskresenskiy AM, Zervos MJ, Lerner SA, Chow JW (2003) Multiplex PCR for Detection of Aminoglycoside Resistance Genes in Enterococci. Antimicrob Agents Chemother 47, 1423–1426. <https://doi.org/10.1128/AAC.47.4.1423-1426.2003>.

Van de Klundert JA, Vliegenthart JS (1993) PCR detection of genes coding for aminoglycoside modifing enzymes. In: Persing DH, Smith TF, Tenover FC, White TJ, editors. Diagnostic molecular epidemiology. Washington: American Society for Microbiology, p. 547-52.

Vankerckhoven V, Van Autgaerden T, Vael C, Lammens C, Chapelle S, Rossi R, Jabes D, Goossens H (2004) Development of a Multiplex PCR for the Detection of *asa1* , *gelE* , *cylA* , *esp* , and *hyl* Genes in Enterococci and Survey for Virulence Determinants among European Hospital Isolates of *Enterococcus faecium*. J Clin Microbiol 42, 4473–4479. <https://doi.org/10.1128/JCM.42.10.4473-4479.2004>.

Weisburg WG, Barns SM, Pelletier DA, Lane DJ (1991) 16S ribosomal DNA amplification for phylogenetic study. J Bacteriol 173, 697–703. <https://doi.org/10.1128/jb.173.2.697-703.1991>.

Yi M, Zou J, Zhao J, Tang Y, Yuan Y, Yang B, Huang J, Xia P, Xia Y (2022) Emergence of *optrA*-Mediated Linezolid Resistance in *Enterococcus faecium*: A Molecular Investigation in a Tertiary Hospital of Southwest China from 2014–2018. IDR Volume 15, 13–20. <https://doi.org/10.2147/IDR.S339761>.

Yoo IY, Kang OK, Shim HJ, Huh HJ, Lee NY (2020) Linezolid Resistance in Methicillin-Resistant *Staphylococcus aureus* in Korea: High Rate of False Resistance to Linezolid by the VITEK 2 System. Ann Lab Med 40, 57–62. <https://doi.org/10.3343/alm.2020.40.1.57>.

Zanella RC, Brandileone MCC, Bokermann S, Almeida SCG, Valdetaro F, Vitório F, Moreira MDFA, Villins M, Salomão R, Pignatari ACC (2003) Phenotypic and Genotypic Characterization of *VanA* *Enterococcus* Isolated During the First Nosocomial Outbreak in Brazil Microbial Drug Resistance 9, 283–291. <https://doi.org/10.1089/107662903322286490>.
